# A new perceptual paradigm to investigate the visual remapping of others’ tactile sensations onto one’s own body shows “mirror touch” for the hands

**DOI:** 10.3389/fpsyg.2014.00095

**Published:** 2014-02-10

**Authors:** Helge Gillmeister

**Affiliations:** Department of Psychology, University of EssexColchester, UK

**Keywords:** visual remapping of touch, mirror touch, perceived intensity, tactile, somatosensory, passive, active

## Abstract

The last two decades have seen a multitude of publications showing the activation of an observer’s somatosensory cortical system during the observation of touch on another person. Behavioral demonstrations of “mirror touch,” however, have been slow in coming forward, and have so far primarily been shown as “visual remapping of touch” on the face. The present study uses a new paradigm to investigate the mirroring of others’ tactile sensations: a 2-AFC task of intensity judgment for touch on the observer’s left and right index finger pads. Observers viewed a left and right hand in an egocentric position, which were either touched passively (pencil moving to touch index finger pad) or actively sought touch (index finger moving to touch pencil). Touch and no-touch events for the two viewed hands were designed to eliminate confounding effects of spatial attention. Felt touches were either concurrent with viewed touch or no-touch events, or were delayed in time to assess potential response bias. The findings demonstrate visual remapping of touch for touch on the hands. If touch was shown on one of the hands only (e.g., left), observers were more likely to perceive touch on the same hand (i.e., their own left hand) as more intense than touch on the other hand even if tactile intensities did not differ, compared to touch shown on both or neither hand. These remapping effects occurred only when viewed and felt touches were concurrent, they were strongly modulated by the way in which viewed touch was incurred, and they were more reliable for touch on the left hand. A second, control experiment, in which touch observation was replaced by bright dots shown on or next to the finger pads, confirmed that these effects were largely due to genuine tactile mirroring rather than to somatotopic cueing. This 2-AFC tactile intensity judgment task may be a useful paradigm to investigate the remapping of others’ tactile sensations onto an observer’s own body.

## INTRODUCTION

“Mirror touch” is the activation of an observer’s somatosensory system, normally recruited during tactile perception, through mere viewing of touch on another person or object (see Keysers et al., 2010, for a recent review). It was first described by Keysers et al. (2004) as an activation specific to secondary somatosensory cortex (SII) regardless of whether touch on a person or an object was observed, and later by Blakemore et al. (2005) as an activation much more specific to touch on a person than on an object and including not only SII but also primary somatosensory cortex (SI) and the premotor, temporal, and parietal regions comprising the human mirror neuron system for action. The process of representing others’ somatic sensations may enable us to know how another person feels and is thought to be essential for developing empathic feelings (e.g., Bufalari et al., 2007).

Mirror touch occurs to different degrees in different people, which is thought to be linked to individual levels of cognitive and affective empathy (Gazzola et al., 2006; Banissy and Ward, 2007; Schaefer et al., 2012). Typically, it does not lead to tactile perceptions in the observer. In 1.6% of people (“mirror-touch synaesthetes”), however, the viewed body of another person may be overincorporated into the observer’s body schema due to an overactivation of the network of frontal and parietal areas that are involved in shared representations and self-other discrimination, respectively (Banissy et al., 2009). Rather than merely using the observed information to understand others’ feelings, mirror-touch synaesthetes may perceive themselves to be the recipients of the observed touch, and thus experience touch on their own body every time they see another person being touched (Blakemore et al., 2005).

If areas of the tactile mirror system become activated by touch observation, even when this activation is below the threshold for conscious perception, it implies that this would increase the sum total of somatosensory activity from afferent input due to touch on the observer’s own body. It has long been known that, compared to a sensory stimulus presented alone, multiple simultaneous stimuli in the same modality improve detection and lower reaction times, presumably due to a process of neural summation (e.g., Raab, 1962).

In a pioneering study, Serino et al. (2008) tested whether the perception of a near-threshold tactile stimulus on the face could be improved by simultaneously viewing a face being touched on a computer monitor. Observers were asked to indicate whether they felt a touch on the left, the right, or both cheeks, while they were viewing a face being either touched on the left, the right, or both cheeks, or merely approached by one or two index fingers from the left, the right, or both sides. Bilateral tactile stimulation, where one cheek was stimulated more intensely than the other, yielded worse performance than unilateral stimulation – akin to extinction phenomena in patients. Interestingly, there was less extinction of the weak tactile stimulus by the strong one when observers viewed touch on a face, compared to when the face was merely approached by one or two fingers. Serino et al. (2008, 2009) suggested that this “visual remapping of touch” (VRT) brought to conscious awareness tactile information that would otherwise not be perceived in non-synaesthetic observers, due to fronto-parietal feedback. Though not explicitly stated, their findings indicate neural summation in the somatosensory system as a result of this feedback.

In a later brain imaging study, Cardini et al. (2011) showed that viewing touch on the face increased activity in the ventral intraparietal cortex and, for some conditions, in the somatosensory cortices, ventral premotor cortex and right insula, compared to viewing a hand merely approaching the face or object, again implying summation of neural activity in some parts of the somatosensory system. The same study also suggests, however, that the neural mechanisms of visuotactile integration that underpin mirror touch are likely to be more complex. Specifically, somatosensory cortical activity increased when viewing touch on another person or object, similar to Keysers et al.’s (2004) original findings, but showed a trend in the opposite direction when viewing one’s own face. Activity in ventral premotor cortex also increased when viewing touch on another person or object, but decreased when viewing touch on oneself.

Over the past few years, studies have begun to identify criteria that constrain or facilitate VRT. For example, VRT was shown to be specific to observing a face compared to a house (Serino et al., 2008), larger if the observed person belongs to the same ethnic or political group as the observer (Serino et al., 2009) and maximal if one’s own face is viewed (Serino et al., 2008; Cardini et al., 2011). Brain imaging showed that watching videos of oneself, another person or an object being touched or just approached by a human finger gives rise to differential activity in the somatosensory cortices, ventral intraparietal, and premotor cortices and right insula (Cardini et al., 2011). Somatosensory cortex is also differentially active as a function of whether viewed touch on a hand is presented from an egocentric or an allocentric perspective (Schaefer et al., 2009). In sum, it is thought that the incorporation of another’s body, which gives rise to mirror touch, is strongest the better the perceived match is between one’s own and an observed body.

The purpose of the present study was to test whether viewing touch modulates other aspects of the tactile experience, and has other limiting factors. Specifically, the perceived intensity of supra-threshold tactile stimuli is hypothesized to increase through neural summation in the somatosensory system via fronto-parietal feedback from viewing touch. For all sensory modalities, the perception of intensity depends directly on the neural activity evoked by a stimulus, and thus on its physical energy. Increasing vibrotactile intensities increases the number of activated SI neurons (Johnson, 1974), and the frequency of their discharge, mirroring the behavior of cutaneous receptors. The perceived intensity of a felt touch should thus increase whenever the somatosensory system is simultaneously engaged in the simulation of a viewed touch. This would be in line with the improved detection of bilateral tactile stimuli from touch observation shown by Serino et al. (2008, 2009) and Cardini et al. (2011), and with the enhancement of neural activity in the somatosensory cortices from viewing touch on another person shown by Cardini et al. (2011).

What factors other than the extent of self-relatedness may limit tactile simulation? Until recently, VRT had only been shown for touch on the face. Brain imaging studies have shown mirror touch for hands (Schaefer et al., 2009, 2012; Pihko et al., 2010) and legs (Keysers et al., 2004), and Banissy and Ward (2007) have reported synaesthetically experienced mirror-touch for both face and hands. Therefore, behavioral effects of tactile simulation in non-synaesthetic observers are unlikely to be limited to the face. Indeed, a recent study showed that VRT can be found for the hands. Cardini et al. (2013) reported that the enhancement of tactile spatial acuity that results from viewing one’s own or others’ hands reduces when the observer’s touched hand and the seen image of it are spatially misaligned. VRT was shown as a restoration of the enhancement effect for misaligned hands when observers viewed a cotton bud touching their hand at the same time, compared to the cotton bud merely approaching the hand. Similar to Cardini et al., the present study shows touch and no-touch stimuli on left and right hands, which are presented from an egocentric perspective, but tests whether the visual touch and no-touch events systematically affect observers’ perceived intensity of felt tactile stimuli on their own hands.

It is also largely unknown whether the type of touch observed modulates tactile simulation. Brain imaging studies have shown that the tactile mirror system is activated differentially depending on the animacy and intentionality of observed touch (Ebisch et al., 2008; Streltsova and McCleery, 2012). The present study asks whether behavioral effects of mirror touch are sensitive to the way in which the observed touch is incurred. Specifically, it compares passively received touch (a pencil touching a fingertip), which is the type of touch typically viewed in studies of mirror touch, to actively sought touch (a finger moving to touch a pencil). If self-relatedness brings about stronger touch mirroring, then effects of tactile simulation might be stronger for passive than active touch viewing because observers themselves are passively touched. On the other hand, the experience of active touch, due to its motor components, activates a larger network of brain areas than passive touch (Simões-Franklin et al., 2011; Ackerley et al., 2012). If this pattern is also present for active and passive touch observation and feeds back to somatosensory regions, these may be more strongly activated and thus lead to more tactile simulation than passive touch.

To demonstrate genuine effects of mirror touch, a perceptual task paradigm must not be vulnerable to contamination from response bias or spatial attention. Furthermore, genuine tactile mirroring should be limited by the temporal proximity of visual and tactile events. To avoid the confounding effects of response bias, the present study presented two simultaneous tactile stimuli, one on each index finger, and gave participants a 2-alternative forced choice (2-AFC) of which touch felt more intense (Gillmeister and Eimer, 2007). Most trials presented same-intensity stimuli, which should yield an approximately equal proportion of left and right hand responses. If the observer views touch on one hand at the same time, it should increase the sum total of neural activity evoked by felt touch on that hand, and therefore increase its perceived intensity, compared to the other hand on which no touch is viewed. The corresponding systematic change in the proportion of left vs. right hand responses may therefore be taken as a behavioral index of mirror touch, or VRT.

Naturally, observing touch on one hand, but not on the other, would draw spatial attention to the hemifield where the touch occurred and thus bias responses toward one’s own hand in this hemifield. If the left hand is chosen as feeling a more intense touch after viewing touch on a left hand, one would be unable to dissociate a purely perceptual effect of simulating left-hand touch from a unilateral response bias as a result of spatial attention to the left side of space. To avoid this confound, in Experiment 1 of the present study a similar-size movement of the pencil or finger always occurred for each of the two hands, sometimes resulting in touch of the finger pad (pencil touching finger or finger touching pencil) and sometimes not (pencil moving into space next to the finger or finger moving into space next to the pencil). In trials in which a touch event occurred on one of the hands, a movement of the pencil, or finger also occurred for the other hand without resulting in touch. Since spatial attention would now not be drawn more to one hemifield than to the other, any remaining VRT effects on the hand may be attributed to genuine tactile simulation.

However, tactile attention can be far more spatially specific than simply to one or the other hemifield (e.g., Eimer and Forster, 2003), and may be drawn toward the specific location of the finger pad for a viewed hand that is touched, and toward the space next to the finger pad for a viewed hand that is not touched. Again, this would render a perceptual account indistinguishable from an attentional or response bias account of any potential VRT effects. To test the contribution of more spatially specific cueing, Experiment 2 showed bright dots on the finger pad or in the space next to the finger pad instead of touch or no-touch events. Banissy et al. (2009) found that, compared to viewing touch, a flash of light on the cheek did not induce synaesthetic experiences in mirror-touch synaesthetes. Similarly, it is hypothesized that, unlike the observation of touch, a bright green dot will not affect the pattern of left vs. right hand responses if the effects of touch observation on perceived tactile intensity are solely attributable to genuine tactile mirroring.

In addition, the present study manipulated the temporal proximity of viewed and felt touches to help dissociate genuine effects of tactile mirroring from response biases induced by the viewed events. For half the trials, the felt and viewed touches were concurrent, and for the other half, the felt touch was delayed by 1000 ms relative to the visual touch event. To equate trial length and to ensure observers would always view the visual touch events, a go/no-go paradigm was used, in which the response (or the withholding of a response) was asked for by a visual event only after the delayed tactile stimulus (or equivalent delay in concurrent touch trials). Meredith et al. (1987) showed that stimuli from different sensory modalities produce enhanced neural responses when their peak discharge periods overlap, which occurs when stimuli are in close temporal proximity and up to within a few hundred milliseconds of one another. Accordingly, perceptual multisensory interactions tend to be stronger the closer in time they are. For example, the facilitatory effects on detection found during bimodal stimulation condition are eliminated when events are offset by 500 ms (e.g., Frassinetti et al., 2002). That is, any genuine effects of mirror touch that are present for concurrent touch trials should be very much reduced, if not eliminated, for trials in which felt touches are delayed by 1000 ms. If visual events merely bias decisions about the perceived intensity of felt touches through somatotopic cueing, however, there should be no difference between effects of viewing touch in concurrent and delayed touch trials.

## MATERIAL AND METHODS

### PARTICIPANTS

Thirty-seven participants took part in Experiment 1 (9 men, 5 left-handed, mean age: 24.1 years). One of them was excluded because their false alarm rate (responses in no go trials) exceeded 20% of trials, and a further participant was excluded because their performance in different intensity trials was at chance. Thirty-six participants took part in Experiment 2 (12 men, 3 left-handed, mean age: 23.1 years). Four of them were excluded because their false alarm rate exceeded 20% of trials. All had normal or corrected-to-normal vision. The study was conducted in accordance with the Declaration of Helsinki (1964) and was approved by the local ethics committee. Informed written consent was obtained from each participant prior to testing.

### MATERIALS

**FIGURE 1:**
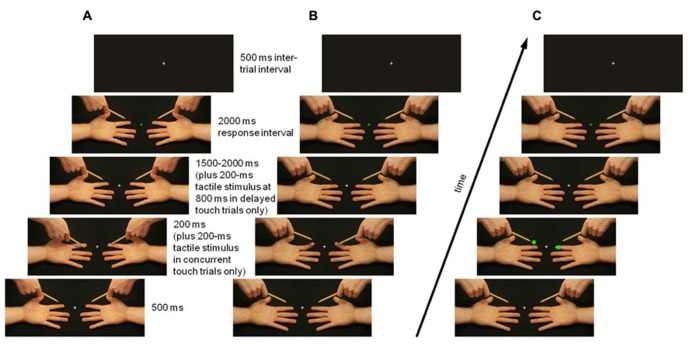
**Experimental design and timing of visual and tactile stimulation in an example trial (touch/dot on right hand, no touch/no dot on left hand).** In Experiment 1, task-irrelevant viewed touch and no-touch events were, in separate blocks, passively received **(A)** or actively sought **(B)**. In Experiment 2, bright green dots were shown on or above the index finger pads instead of touch or no-touch events **(C)**. Replacement of neutral with touch or no-touch images, and vice versa, gave the impression of apparent motion of the pencil [passive touch trials **(A)**] or finger [active touch trials **(B)**] resulting in touch or no touch. Task-relevant tactile stimuli were presented to the observer’s left and right index finger pad either simultaneously with each touch/no-touch/dot image (concurrent touch trials) or 1000 ms later (in delayed touch trials). Observers fixated on a white central cross, which changed color at the end of the trial to indicate whether the intensity judgment response should be given (green) or withheld (red).

A Dell Optiplex GX230 with a 24′′ monitor was used to present visual and tactile stimuli. Visual stimuli measured 40.8° of horizontal and 15.5° of vertical visual angle, and were presented against a black background in the center of the computer screen. The neutral hands image showed the left and right hand in a supine position with a pencil held above each (see **Figure 1**). In Experiment 1, touch images showed the pencil lower down, depressing one or both hands’ index finger pads (passive touch trials; see **Figure 1A**) or they showed one or both hands’ index fingers higher up, pressing against the pencil (active touch trials; see **Figure 1B**). No-touch images showed the pencil lower down, in a position next to one or both hands’ index fingers (passive no-touch trials; see **Figure 1A**) or they showed one or both the supine hand’s index finger higher up, next to the pencil (active no-touch trials; see **Figure 1B**). In Experiment 2, neutral hands images were shown throughout the trial, and instead of pencil or finger displacements, bright green dots appeared on or above the index finger pads, at the positions where the touch or no-touch event occurred in active touch trials in Experiment 1 (see **Figure 1C**). Superimposed on all images was a white central fixation cross (0.8° × 0.8°). At the end of each trial, this cross turned green in go trials in which an intensity judgment response was required, or red in no-go trials in which the response should be withheld.

A tactile controller and mechanical tactile stimulators (Heijo Research Electronics, London, UK) were used to deliver tactile stimuli to participants’ left and right index fingers in each trial. Stimulators were solenoids that drove a blunt plastic tip against the index finger pad whenever a current was passed through them. The strength of the current was either weak or strong on both fingers (in same intensity trials) or weak on one finger and strong on the other (in different intensity trials). White noise was played through in-ear headphones to mask any sounds made by the solenoids.

Responses (brief lifting of the left or right hand) were recorded and timed with a custom-built infrared-barrier device. A 1000-Hz sinusoidal tone was played through the headphones if the infrared beam was not disrupted by a lifting hand in go trials, and when it was disrupted mistakenly in no-go trials.

### DESIGN AND PROCEDURE

Participants sat in a semi-darkened room with their hands palm-up on a table top so as to mimic the position of the hands depicted on-screen. Tactile stimulators were attached to their left and right index finger pads with medical tape. To prevent participants from viewing their own hands, they were placed under a board covered with a black cloth.

Participants were asked to pay close attention to the touches felt on their own hands and in each trial to decide whether the left or right hand touch felt more intense. They were asked to monitor the fixation cross on the screen and make a response (brief lifting of the left hand if the left touch felt more intense, or of the right hand if the right touch felt more intense) when the fixation cross turned green, and to refrain from responding if it turned red.

Experiment 1 consisted of ten blocks of 48 trials, five blocks showing passive touch/no-touch images (see **Figure 1A**) and five showing active touch/no-touch images. Passive and active blocks were presented alternately, with the order counterbalanced across participants. Each trial began with the 500-ms presentation of a neutral hands image, which was then replaced by a touch or no-touch image. There were four such images: touch on both hands (touch both), no touch on either hand (touch none), touch on the right hand and no touch on the left hand (touch right; see **Figure 1**), and touch on the left hand and no touch on the right hand (touch left), presented in equal proportions and randomly intermixed within a block.

Touch or no-touch images were shown for 200 ms. In concurrent touch trials, images were accompanied by 200 ms tactile stimuli to the participant’s index finger pads. Then, the neutral hands image was presented again for a random duration between 1500 and 2000 ms. In delayed touch trials, 200 ms tactile stimuli were delivered to the participant’s index finger pads between 800 and 1000 ms into the presentation of the neutral hands image, that is, 1000 ms after the onset of the presentation of the touch or no-touch image. Half of all trials were concurrent touch trials, and the other half were delayed touch trials, randomly intermixed within a block.

At the end of each trial, the white fixation cross superimposed in the neutral hands image changed color for 2000 ms, indicating that a response was required (green; 40 trials per block) or should be withheld (red; 8 trials per block). A tone was played for 300 ms if a response was not made within the 2000-ms window in go trials and if a response was made in no-go trials. During the 500-ms intertrial interval, an image of an off-black rectangle with a central white fixation cross was shown.

Experiment 2 consisted of five blocks of 48 trials, composed in the same way as in Experiment 1, except that instead of touch or no-touch images, one of four possible dot or no dot images was shown: dots on both hands (dot both), dots above both hands (dot none), dot on the right hand, and dot above the left hand (dot right; see **Figure 1**), and dot on the left hand and dot above the right hand (dot left), presented in equal proportions.

In both experiments, most trials (40 per block) presented tactile stimuli of the same intensity to both hands, but some trials (8 per block) presented a weak touch to one hand and a strong touch to the other. These trials were included to check that participants were following task instructions, and were used to present performance feedback (accuracy of intensity judgment) after each block.

## RESULTS

### EXPERIMENT 1

Performance in different intensity trials was 91.7%, suggesting that participants followed task instructions well. A repeated-measures ANOVA for the within-subject factors action (passive vs. active touch trials) and delay (concurrent vs. delayed touch trials) showed that performance was better in delayed touch trials (92.9%) than in concurrent touch trials (90.4%) [*F*(1,34) = 7.0, *p* = 0.012], but there was no difference between passive (91.5%) and active (91.9%) touch blocks [*F*(1,34) < 1, *p* = 0.784]. The interaction between delay and action was marginally significant [*F*(1,34) = 3.6, *p* = 0.065], and indicated that performance improvement in delayed touch trials was present in active touch blocks [*F*(1,34) = 10.0, *p* = 0.003] but absent in passive touch blocks [*F*(1,34) < 1, *p* = 0.521].

**FIGURE 2:**
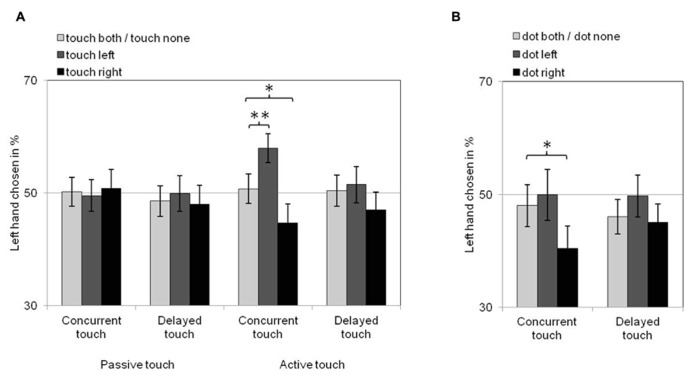
**Visual remapping of touch effects for Experiment 1 (A) and Experiment 2 (B).** Bars show the proportion of trials in which the left hand was chosen as feeling the more intense touch as a function of observing touch **(A)**/dots **(B)** on the left hand (dark gray bars), on the right hand (black bars) or on both or neither hands (light gray bars) for all conditions of action [**(A)** only] and delay. Error bars denote standard error of the means. These proportions are around chance (50%) when observing touch/dots on both hands or none. Proportions *above* those reflect VRT effects from observing *left* hand touch/dots (dark gray bars) and proportions *below* those reflect VRT effects from observing *right* hand touch/dots (black bars). Single asterisks denote significant pairwise comparisons at the 0.05 level. Double asterisk indicates significant pairwise comparison at the 0.005 level.

Choices of which hand felt the more intense touch in same-intensity trials can be seen in **Figure 2A**. When touch on both hands or on neither hand were observed, choices between the left and the right hand were around chance (50%). Observers were more likely to choose one of the hands in trials in which only one of the hands was seen to receive touch. Specifically, they were more likely to choose their left hand as having felt the more intense touch when they viewed touch on the left hand, and to choose their right hand as having felt the more intense touch when they viewed touch on the right hand. This tendency was only present in active touch blocks and it was particularly pronounced when felt touches were concurrent with observed touches.

For all trials in which the left (right) hand was chosen as having felt the more intense touch, statistical analyses compared the frequency of trials in which touch on both hands or touch on neither of the hands was observed (collapsed data) to the frequency of trials in which touch was observed on the left (right) hand. Comparisons were made in separate repeated-measures ANOVAs for the within-subject factors touch (touch both/none vs. touch left; touch both/none vs. touch right), action (passive vs. active touch trials), and delay (concurrent vs. delayed touch trials). For trials in which the left hand was chosen as feeling the more intense touch (see **Figure 2A**, dark gray bars vs. light gray bars), Bonferroni-adjusted planned pairwise comparisons of the estimated marginal means of trial frequencies for each combination of touch, action, and delay showed that the left hand was chosen significantly more often when touch on the left hand was observed than when touch on both hands or none was observed only when the felt tactile stimulus was concurrent with the viewed active touch [*F*(1,34) = 11.0, *p* = 0.002, $\eta_{p}^{2}$ = 0.245] but not when it was delayed [*F*(1,34) < 1, *p* = 0.600, $\eta_{p}^{2}$ = 0.008] or when (concurrent or delayed) passive touch was viewed [*F*(1,34) < 1, *p* ≥ 0.561, $\eta_{p}^{2}$ ≤ 0.010].

A similar pattern was found in the planned pairwise comparisons for trials in which the right hand was chosen as feeling the more intense touch (see **Figure 2A**, black bars vs. light gray bars). The right hand was chosen significantly more often when touch on the right hand was observed than when touch on both hands or none was observed only when the felt tactile stimulus was concurrent with the viewed active touch [*F*(1,34) = 5.5, *p* = 0.025, $\eta_{p}^{2}$ = 0.139] but not when felt touch was delayed [*F*(1,34) = 2.0, *p* = 0.171, $\eta_{p}^{2}$ = 0.054] or when (concurrent or delayed) passive touch was viewed [*F*(1,34) < 1, *p* ≥ 0.797, $\eta_{p}^{2}$ ≤ 0.002].

### EXPERIMENT 2

Performance in different intensity trials was 91.7%, indicating that task instructions were followed. A repeated-measures ANOVA for the within-subject factor delay (concurrent vs. delayed touch trials) found no difference between concurrent (91.0%) and delayed touch trials (92.5%) [*F*(1,31) < 1, *p* = 0.409].

Choices of which hand felt the more intense touch in same intensity trials are displayed in **Figure 2B**. For all trials in which the left hand was chosen as feeling the more intense touch (see **Figure 2B**, dark gray bars vs. light gray bars), and unlike Experiment 1, choices between the left and the right hand were around chance (50%) even in trials when a dot on the left hand was observed. For trials in which the right hand was chosen (see **Figure 2B**, black bars vs. light gray bars), the right hand was chosen more often when a dot on the right hand was observed when the felt tactile stimulus was concurrent with a dot on the right hand.

These observations were confirmed by Bonferroni-adjusted planned pairwise comparisons of the estimated marginal means of trial frequencies for each combination of the within-subject factors dot (dot both/none vs. dot left; dot both/none vs. dot right) and delay (concurrent vs. delayed touch trials) in separate repeated-measures ANOVAs for all trials in which one or the other hand was chosen. These analyses showed that observing a dot on the left hand did not result in the left hand being chosen more often than observing dots on both hands or none, both when the felt tactile stimulus was concurrent with the viewed dots [*F*(1,31) < 1, *p* = 0.509, $\eta_{p}^{2}$ = 0.014] and when it was delayed [*F*(1,31) = 2.9, *p* = 0.097, $\eta_{p}^{2}$ = 0.086]. For trials in which the right hand was chosen as feeling the more intense touch, however, there was a significantly higher number of trials in which a dot on the right hand was observed than trials in which a dot on both hands or none was observed only when the felt touch was concurrent with the viewed dot [*F*(1,31) = 5.3, *p* = 0.028, $\eta_{p}^{2}$ = 0.146] but not when it was delayed [*F*(1,31) < 1, *p* = 0.703, $\eta_{p}^{2}$ = 0.005].

## DISCUSSION

The aim of this study was to test whether forced-choice intensity judgments for touch on the hands would be systematically modulated by viewing touch on another person’s hands, whether this was sensitive to the way in which the viewed touch was incurred, and to what extent modulations could be explained as genuine perceptual-level effects of tactile simulation or as response bias from somatotopic cueing. The results suggest that observed touch on another person’s hand is remapped onto the somatosensory representation of one’s own hand. Specifically, observers perceived a touch on their own hand as more intense if it was accompanied by a viewed touch on the equivalent hand of another person. Similar to Cardini et al.’s (2013) recent study, this shows that perceptual effects of mirroring the tactile sensations of another person are not limited to the face, but can also be found for the hands.

Visual remapping of touch in non-synaesthetic observers has previously only been shown using a detection paradigm adapted from studies in patients with tactile extinction phenomena (e.g., Serino et al., 2008, 2009) or in terms of a modulation of visual enhancement of touch (Cardini et al., 2013). In Serino et al.’s (2008, 2009) typical VRT paradigm observers are asked to indicate whether they felt a touch on the left, right or both cheeks, and accuracy in reporting the presence of two touches (rather than the stronger of the two only) improved during the observation of touch. In Cardini et al.’s paradigm, the concurrent observation of touch on the hand was found to improve accuracy in reporting the spatial discrimination of tactile gratings in a condition in which such enhancement is not normally present. The present study shows for the first time that the perception of stimulus intensity can also be modulated by viewing touch on another person’s body. In this paradigm observers made a forced choice about which of two (mostly equally intensive) touches on the hands felt more intense, and the proportion of times each hand was chosen was affected systematically by the observed visual touch events.

Experiment 1 found that observers were more likely to chose the left (right) hand as feeling the more intense touch when they observed touch on the left (right) hand, together with a no-touch event (pencil or finger moving but not touching) on the other hand, compared to when they observed touch on both of the hands or neither of the hands. This was specific to the observation of actively sought, concurrent touch. As a similar-size apparent movement always occurred for each of the two hands, sometimes resulting in touch of the finger pad and sometimes not, it can be ruled out that these effects are due to a mere drawing of (visual) spatial attention to one or the other hemispace by the touch, compared to the no-touch, stimulus. The contribution of visual-spatial attention to the facilitation of tactile detection, especially from more eccentric visual events, has previously been shown, although this was argued to only occur in the absence of a body part (Serino et al., 2008).

Related to this, left and right viewed touch in Serino et al.’s (2008, 2009) studies would have introduced a spatial bias because one finger moved onto the observed cheek (or next to it), while the other finger stayed at the bottom of the screen. For the interpretation of Serino et al.’s (2008, 2009) findings this is not problematic, because detection in bimodal trials was found to be facilitated only in trials in which the finger moved onto the observed cheek, but not when the finger moved next to it, and because facilitation was indiscriminate, rather than specific for the side of the occasionally extinguished weaker tactile stimulus. However, future studies, especially those looking more closely at the effects of somatotopy in VRT phenomena, may consider eliminating hemispatial bias on a trial-by-trial basis like in the present study by pairing touch events in one hemispace with no-touch events that draw spatial attention to a comparable degree in the other hemispace.

Experiment 2 was designed to measure the contribution of somatotopic cueing – spatial attentional cueing toward the specific location of the finger pad – by showing bright dots on the finger pad or in the space next to the finger pad instead of touch or no-touch events. Here, observers did not choose the left hand as having felt the more intense touch any more often when they observed a bright dot on the left finger pad (and a bright dot above the right finger pad), compared to when they observed bright dots on both or neither finger pads. This is similar to the absence of a synaesthetic experience of touch when mirror-touch synaesthetes are shown a flash of light instead of a touch on another person’s face (Banissy et al., 2009). For choices made about the right hand, however, there was some evidence of a tendency to choose this hand as having felt the more intense touch more often when a dot on the right finger pad was observed than when a dot on both or neither finger pads were observed. This suggests that, for the right hand, but not for the left, somatotopic cueing alone can bring about some VRT-like effects.

The proposed mechanism of these VRT effects of tactile simulation, specifically those observed for the left hand, is that the seen touch increases the amount of activity in the somatosensory system via feedback from frontal-parietal mirror networks, and therefore increases the perceived intensity of a felt touch on a corresponding body part via neural summation. Although it is likely that multisensory processes other than neural summation contribute to the full picture of mirror touch phenomena, a neural summation account is in line with the improved detection of bilateral tactile stimuli from touch observation shown by Serino et al. (2008, 2009) and Cardini et al. (2011), and with the enhancement of neural activity in the somatosensory cortices from viewing touch on another person shown by Cardini et al. (2011). Furthermore, the present results also suggest that the VRT effects for the perception of tactile intensity are limited to the observation of actively sought touch and that, for the right hand/left hemisphere, they may be subject to response bias from somatotopic cueing. In the following each of these points shall be considered in turn.

### PERCEPTUAL EFFECTS OF MIRROR TOUCH ARE STRONGER FOR ACTIVELY SOUGHT THAN PASSIVELY RECEIVED TOUCH

The present study shows that behavioral effects of mirror touch are sensitive to the way in which the observed touch is incurred. It was found that the perception of tactile intensity on the hands is modulated only by viewed touch that is actively sought (a finger moving to touch a pencil), but not by touch that is passively received (a pencil moving to touch a finger). The absence of perceptual effects for passive touch observation contrasts with Serino et al.’s (2008, 2009) demonstrations of the visual remapping of passively received touch on the face. The sight of passive touch would have been far more similar to what the observer himself experienced. It might be argued that passive touch was therefore more self-related than active touch in this experiment, and should thus have been incorporated more strongly. This was clearly not the case, and may reflect inherent differences between the potential self-relatedness of face vs. hand stimuli.

What other factors might have been at play to give rise to these differing patterns of results for passive touch observation on face and hands? One is the possibility that tactile detection of weak stimuli as measured in Serino et al.’s (2008, 2009) extinction paradigm is more sensitive to perceptual effects of mirror touch than the present 2-AFC tasks of perceived intensity. Another is that presenting video stimuli at the location of the touched hand, as done by Serino et al. (2008, 2009), is more effective at eliciting mirror touch than the apparent motion induced by the images presented on a computer screen in the present study. A third possibility is that perceptual effects of mirror touch may generally be stronger for the face than for the hands. This may be because tactile stimulation of the face is potentially more harmful to the organism, and thus seen as more threatening, than tactile stimulation of the hand. If it can enable one to avoid harm, it would be advantageous to mirror sensory events on the face more than those on the hands. This would explain why it appears as though only the observation of a face elicits perceptual effects of mirroring passively received tactile sensations.

It is most likely, however, that VRT mechanisms are slightly different for touch on hand and face since their tactile experience differs. For the face, passive touch observation may potentially elicit stronger mirroring than active touch observation because the face typically receives passive touch. For the hand, which is far more engaged in active exploration of the world, active touch observation may elicit stronger mirroring than passive touch. Although several brain imaging studies have shown mirror touch during the observation of passive touch on the hands (Bufalari et al., 2007; Ebisch et al., 2008; Schaefer et al., 2009, 2012; Pihko et al., 2010), it is interesting to note that some of these studies introduced an additional element of active touch as the touching action was performed by another hand (Ebisch et al., 2008; Pihko et al., 2010), similar to Serino et al.’s (2008, 2009) studies in which one or two hands moved toward the face. It is likely that the additional element of active touch increases the measured effects of tactile mirroring.

Active touch, due to its motor component, has been shown to activate frontal, sensorimotor and primary somatosensory regions more than passive touch (Simões-Franklin et al., 2011; Ackerley et al., 2012). This may mean that the observation of active touch also engages frontal, motor regions more than the observation of passive touch, and thus leads to greater activity in somatosensory regions via feedback links with these areas. This may either enhance sensorimotor simulations in strength, or prolong them in time, leading to more easily measurable effects on perception than mere passive touch. This is in line with the present pattern of stronger VRT effects for active compared to passive touch observation, but also suggests that VRT effects in the passive touch condition may not have been measured because, even though another hand was visible, the viewed touch itself was produced by a pencil and so the visual stimulus could not have conveyed the feeling of touch by the touching hand in addition to that felt by the receiving hand.

### PERCEPTUAL EFFECTS OF MIRROR TOUCH ARE STRONGER FOR THE LEFT THAN FOR THE RIGHT HAND

VRT-like effects can be considered genuine perceptual-level effects of tactile simulation if it can be shown that they are systematically modulated by the temporal proximity of visual and tactile events. This is because the neural activation profiles of nearer-simultaneous stimuli are more likely to overlap, and such stimuli are therefore more likely to interact perceptually than stimuli that are more temporally separated (e.g., Meredith et al.,1987; Frassinetti et al., 2002).

The present study found that perceived tactile intensity was affected by viewed touch only when viewed and felt touch were concurrent, but not when felt touch was delayed by 1000 ms. Interestingly, the statistical evidence for this was stronger for the left than for the right hand. For the left hand, perceptual effects of mirror touch were present only when the felt touch was concurrent with the observed (active) touch event, and were significant at the 0.005 level with a good effect size (25% of variance explained). For the right hand, VRT effects were also present only for concurrent (active) touch, but, unlike for the left hand, they were significant only at the 0.05 level with an effect size of about half that found for the left hand (14% of variance explained). This shows that the systematic modulation of perceived intensity by viewed touch in this study largely reflects genuine perceptual-level effects of mirror touch, but also suggests that, compared to the left hand, mirror touch on the right hand may in addition be somewhat more susceptible to response biases such as those introduced by somatotopic cueing. Indeed, Experiment 2 found that replacing touch and no-touch events with bright dots on or next to the viewed fingers gave rise to moderate (significance at the 0.05 level, 15% of variance explained) VRT-like effects for the right hand, but not for the left hand.

A hemispheric asymmetry with respect to mirror touch and its perceptual consequences has so far not been reported. The left hand/right hemisphere may be somewhat more optimal for processing tactile signals, and thus less susceptible to response bias. While there are many studies showing equivalence in performance for the left and right hand in a wide variety of tactile and haptic tasks, the few that do show an asymmetry show a left-hand rather than a right-hand superiority (for reviews, see Summers and Lederman, 1990; Fagot et al., 1997). There is a relatively robust left-hand advantage for haptic form recognition (Summers and Lederman, 1990; Fagot et al., 1993), and for discrimination of kinaesthetic information (e.g., Roy and McKenzie, 1978) and spatial orientation (e.g., Benton et al., 1973). There are somewhat less robust findings showing a left-hand advantage for sensitivity to pressure and vibration (e.g., Ghent, 1961).

The left hand/right hemisphere may also be more optimal for integrating visual and tactile information. Longo et al. (2012) found spatial compatibility effects between visual and tactile events at the fingers when the left hand was viewed, but not when the right hand was viewed. They suggested that this laterality effect is linked to processing in right-hemisphere regions such as posterior parietal cortex and the temporo-parietal junction, which are strongly implicated in processing spatial aspects of body-related information, perspective taking, and self-other discrimination. Finally, the right hemisphere, which shows a general dominance for emotional processing (e.g., Natale et al., 1983), may be better optimized for processing the sensorimotor aspects of empathy that are needed for unconscious mimicry and the ability to share the feelings of others (e.g., Leslie et al., 2004).

To summarize, the available evidence suggests that the left hand/right hemisphere may perhaps be more apt to convey the perceptual-level visual-tactile interactions that underlie mirror touch in order to understand the somatic sensations of others, and, due to its higher tactile precision, less susceptible to post-perceptual bias.

In conclusion, the present study introduces a novel paradigm to effectively study the perceptual consequences of mirror touch free from the confounds of spatial attention. The above discussion of the findings highlights several aspects of mirror touch that are worthwhile considering further, such as the systematic investigation of hemispheric asymmetries, the somatotopic correspondence between felt and viewed touches, and the relative contributions of active and passive aspects of viewed touch (touch felt through being touched vs. touch felt through the act of touching).

## Conflict of Interest Statement

The authors declare that the research was conducted in the absence of any commercial or financial relationships that could be construed as a potential conflict of interest.
